# Are curriculum changes the ideal method for increasing undergraduate exposure to tomorrow’s specialties?

**DOI:** 10.2147/AMEP.S81603

**Published:** 2015-03-10

**Authors:** Savan Shah

**Affiliations:** Imperial College, London, UK

**Dear editor**

Smaller specialties such as dermatology, psychiatry, and radiology have often received less teaching time within the undergraduate medical curriculum.^1^ Some critics argue that this may be responsible for the low intake rates within postgraduate training for these specialties. The Royal College of Psychiatrists reported that only 83% of core training post vacancies were filled after two rounds of recruitment in 2012.^2^ Furthermore, the National Health Service identified a shortfall of 220 interventional radiologists in the UK.^3^ Similar workforce problems have been encountered in the USA.^4^ It has been suggested that increasing exposure to these specialties in medical school may allow students to gain a better insight into the specialty and consider it as a viable future career choice.^1^ Although incorporating more dedicated teaching time for small specialties within the curriculum may help to meet future workforce demands, there are strong arguments against implementing it. First of all, teaching time would have to be taken away from the core specialties to accommodate more teaching for smaller specialties. However, knowledge and skills within the core specialties is more important for building a strong foundation for any future career choice. There is no robust evidence to suggest that students would still receive adequate training in core specialties if smaller specialties are given more time. Second, the overall workforce requirements are much greater for the core specialties and the majority of graduates are more likely to enter the core rather than the smaller specialties.

Perhaps a different approach is required. In order to tackle the current shortfall in psychiatrists, the Royal College of Psychiatrists has adopted a particularly strong strategy.^2^ This includes setting up/supporting medical school societies, regular career fairs, annual summer schools, intercalated Bachelor of Science programs, and student elective modules to engage and educate medical students. In addition, financial incentives (including bursaries/grants for conferences, electives, and research, essay prizes, and fellowships) have been used to promote and reward student activity across the variety of psychiatric subspecialties. Although these methods may not have the direct impact of dedicated teaching, they provide ample opportunity for students to explore the smaller specialties without detracting unnecessarily from the core curriculum.
